# Detecting the functional interaction structure of software development teams

**DOI:** 10.1371/journal.pone.0306923

**Published:** 2024-10-24

**Authors:** Christian Zingg, Alexander von Gernler, Carsten Arzig, Frank Schweitzer, Christoph Gote

**Affiliations:** 1 Chair of Systems Design, ETH Zurich, Zurich, Switzerland; 2 genua GmbH, Kirchheim bei München, München, Germany; 3 Complexity Science Hub, Vienna, Austria; 4 Data Analytics Group, Department of Informatics, University of Zurich, Zurich, Switzerland; The University of Jordan Faculty of Business: The University of Jordan School of Business, JORDAN

## Abstract

The functional interaction structure of a team captures the preferences with which members of different roles interact. This paper presents a data-driven approach to detect the functional interaction structure for software development teams from traces team members leave on development platforms during their daily work. Our approach considers differences in the activity levels of team members and uses a block-constrained configuration model to compute interaction preferences between members of different roles. We apply our approach in a case study to extract the functional interaction structure of a product team at the German IT security company *genua GmbH*. We validate the accuracy of the detected interaction structure in interviews with five team members. Finally, we show how our approach enables teams to compare their functional interaction structure against synthetically created benchmark scenarios. Specifically, we evaluate the level of knowledge diffusion in the team and identify areas where the team can further improve. Our approach is computationally efficient and can be applied in real-time to manage a team’s interaction structure. In summary, our approach provides a novel way to quantify and evaluate the functional interaction structure of software development teams that aids in understanding and improving team performance.

## 1 Introduction

Designing and maintaining an efficient organisational structure is essential for highly performant software development teams [1–6]. This is especially the case in agile software development teams which—similar to Open Source Software teams [7]—have a strong focus on self-organisation and organisational flexibility [8]. The key concept behind agile software engineering is a high level of adaptivity, continuous evolution, and flexibility to changes in requirements [9]. As a consequence, the real interaction structure of such teams changes over time to adapt to new challenges. As such, it deviates from the team’s original organisational structure to a new *unknown* one [10–12].

Not knowing the team’s real interaction structure can have a broad range of negative consequences for the team. In the best case, it leads to reduced productivity or a decrease in software quality due to less well-managed and, therefore, less efficient information exchange [13–15]. However, in the worst case, it can result in the undetected emergence of developers possessing mission-critical *unshared* knowledge—e.g., *lone wolfs*, *bottlenecks*, or *organisational silos* [2, 14, 16]—which can have a devastating impact when they leave the team [17].

Despite its importance, the question of how to quantitatively and efficiently derive and evaluate a team’s functional interaction structure based on real observed interactions remains open. Closing this gap would enable teams to detect when employees with a certain role isolate themselves, resulting in organisational silos [18, 19]. In addition, teams could assess and manage their functional interaction structure in real-time and evaluate the effectiveness of team management approaches like Scrum [20], Kanban [21], Extreme Programming [22], or DevOps [23].

In this paper, we provide a method to detect the functional interaction structure of a team and apply it to analyse the interactions from a product team at *genua GmbH*, a German IT security company. We make the following contributions:

- We mine all actions the product team’s members leave on their issue tracker, code review platform, and version control system. We further identify a set of practices allowing us to derive the corresponding interactions between team members, yielding multi-edge interaction networks for each platform.
- To understand the functional interaction structure of a software development team, we need to map the observed interactions between team members to interactions between roles. Traditional social network analysis achieves this by aggregating edges between roles based on their counts or applying statistical models such as the stochastic block model (SBM). However, we show that these approaches are insufficient for our data, as they fail to account for the heterogeneity in the activity of both the roles and the individual team members.
- Instead, we propose a novel method based on a *block-constrained configuration model* (BCCM) [24] that accounts for each team member’s unique capacity to initiate and receive (directed) interactions. Our method allows us to *quantify* the team’s functional interaction structure on each development platform individually, as well as across all platforms.
- We validate the extracted interaction structure through semi-structured interviews with five team members from the product team at *genua*. Using the information obtained from the interviews, we further extend the extracted interaction structure with meta-information on each observed type of interaction. As a result, we obtain the team’s organigraph [25], visualising how different *roles* in a team functionally work together.
- Finally, we show how our block model approach can also be used to compare the knowledge diffusion in the observed interactions with two other hypothetical scenarios. We find that the team currently achieves knowledge diffusion in the upper third of the possible range. Our analysis further shows that extending the agile methods employed by the team is the most promising way to improve knowledge diffusion further.

## 2 Related work

This paper analyses the interaction structure of a software development team with the aim of detecting functional relations between team members of different roles. We split the discussion of the related work into two parts. Focussing on software development, we first review other works analysing the interaction structure of teams. We then explore methods used to detect functional interaction structures in other scientific disciplines.

### 2.1 Analysing the interaction structure of teams

The interaction structure of teams has been studied and characterised in a broad range of empirical studies. Commonly, this is achieved via a network approach. Here, researchers represent individuals as nodes and their interactions as edges. They then compute various network measures, such as the degree distribution, clustering coefficient, or betweenness centrality, to characterise their interaction structure [26–29]. For example, the betweenness centrality could reveal hubs in open-source software teams who route the information flow from peripheral developers into the core team [12]. Entropy measures could show that humans interact with a broad range of peers in the early stages of group formation but narrow down their contacts as time proceeds [30]. Similarly, the *potentiality*, an entropy-based measure, was used to quantify the distribution of interactions across a team [29], thereby proxying the resilience to forming knowledge islands. Using the degree assortativity and clustering coefficient, the impact of the departure of a core developer on an Open Source team was measured [31]. In a similar approach, the authors of [15] studied how well various network measures predict the risk of introducing software defects. Using non-network approaches, the authors of [32] characterised the interaction structure spatially by detecting locations in an office building where employees frequently interact. In other literature, the task redistribution between software developers was studied with agent-based models [33, 34]. However, to the best of our knowledge, no study has yet quantified the functional interaction structure of software development teams based on the roles of their members—a gap we address in this paper. Instead, previous studies in the empirical software engineering literature have focused on the interactions between team members on a per-individual basis. However, similar questions are common in other scientific disciplines, such as biology.

### 2.2 Functional interaction structure detection in biology

In biology, detecting the functional interaction structure is a frequently asked question. For example, researchers study the specialisation in plant-pollinator relations [35, 36], or the tendency of specific types of proteins to react more frequently with each other than expected at random [37]. Finding such functional relations is crucial to understand the dynamics of ecosystems and to predict their behaviour under different conditions. To derive them from the observed interactions in specific systems, several methods have been developed, which—in contrast to the approaches in software engineering—explicitly account for the heterogeneity in the activity levels of the interacting species. For example, the authors of [38] propose a network-theoretical framework allowing to detect over-/underproportional interactions among plant species. Similarly, the authors of [39] study the differences in the structure of a food web across multiple habitat types by aggregating observed interactions using a count-based approach. However, these straightforward methods fail when the activity levels of the interacting species are heterogeneous. The authors of [40] show that simple approaches such as a counting-based approach fail when activity is unevenly distributed across species. This is the case, e.g., in habitats with low activity or for rare species. To counter this, the stochastic block model (SBM)—which we will also use in our work—has been applied [41–43]. The SBM has further been extended to address other original limitations, e.g., cases in which individuals of a species have different activity levels [24, 44], or individuals belong to multiple species [45]. Our study will build on these approaches to address the unique challenges of identifying the functional interaction structure of software development teams.

## 3 Data

In this paper, we study the case of software development in a product team at *genua*. To this end, in Section 3.1, we first introduce the four roles all members of the product team are subdivided into. In Section 3.2, we then discuss how we mined interaction networks from *genua*’s development platforms.

### 3.1 Roles

Based on their tasks and responsibilities, *genua* classifies the members of the product team into four roles:

1. Developers develop, review, and integrate code and changes to implement new features and fix bugs.
2. Documenters write and maintain the user manual and release notes of the product.
3. Product Owners coordinate the team and are responsible for scheduling and prioritising issues.
4. Stakeholders only have a peripheral role within the team. The majority are customer-facing, selling the product to new clients, maintaining it on their sites, or training their internal staff regarding its use. Others perform quality assurance and application testing. Finally, some work on other internal projects adjacent to the product.

We refer to the set of roles as R. To obtain the roles for all team members and years, we followed a two-step process. First, we created lists of all team members active within a given year. Then, we iterated through these lists with two long-term team members to identify each member’s correct role. In rare cases where the two team members were uncertain regarding a role, they contacted additional team members more familiar with the respective case. The resulting data set contains (i) team members’ IDs and (ii) their roles for (iii) each year. We provide summary statistics for this data set in Table 1.

**Table 1:** Summary statistics on team members and interactions for the four roles.

	Team members		Interactions	
	Total	Per year	Total	Per year
Developers	67	30–51	483,878	33,108–73,928
Documenters	8	3–6	19,885	180–3,614
Product Owner	5	1–3	18,888	372–5,751
Stakeholders	62	18–40	21,451	551–5,650

### 3.2 Interaction networks

The product team uses three different platforms to track their work. An *issue tracker* is used to manage and discuss implementations of issues, i.e., bug fixes or feature requests. When new code to resolve an issue is developed, this is tracked on the team’s *code review* platform. Finally, the team employs Git as *version control system* to collaborate on the codebase. As discussed in the following, for each platform, we mined pseudo-anonymised data capturing all *actions* performed by team members. In addition, we identified practices to extract the *interactions* corresponding to these actions and to represent them as networks that capture interaction patterns in the whole team. We provide summary statistics for the resulting networks on all platforms in Table 2.

**Table 2:** Summary statistics on team members and interactions for the three development platforms.

	Team members		Interactions	
	Total	Per year	Total	Per year
Issue tracker	118	44–68	77,616	3,662–15,745
Code review platform	65	17–28	93,256	4,759–14,571
Version control system	75	34–57	101,179	8,152–18,093

#### 3.2.1 Issue tracker

**Fig 1:**
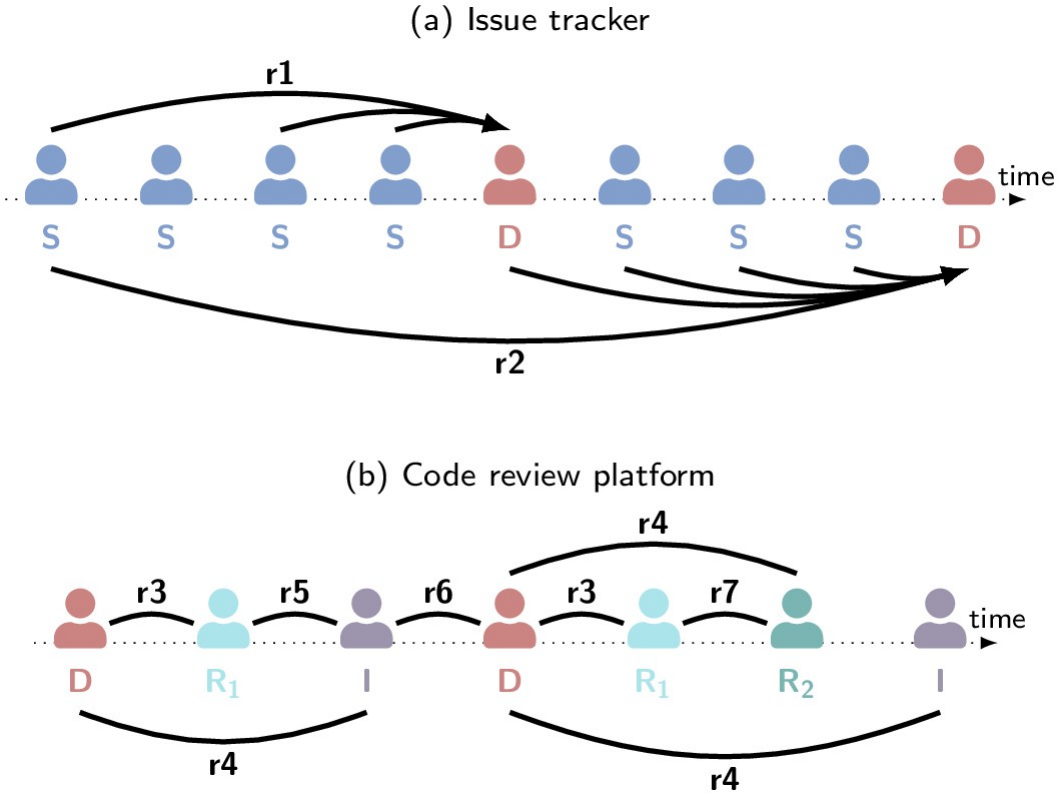
Visualisation of the identified practices r1–r7 to derive interactions. For descriptions of the practices, we refer to the text. (a) Directed interactions derived from an exemplary discussion thread between two team members *S* and *D* on the issue tracker. For clarity, we only show the interactions derived for *D*. (b) Undirected interactions derived from an exemplary change development on the code review platform. On this platform, team members develop *D*, review *R*, or integrate *I* changes. In the example, the change is reviewed by two separate team members, *R*_1_ and *R*_2_. As indicated by the colours, the developer, reviewer, and integrator must be different team members.

The team uses the tool Redmine [46] as their issue tracker. Similarly to an online forum, Redmine maintains separate discussion threads for all issues. In Fig 1a, we show an example of a discussion thread in which two team members, a stakeholder *S* and a developer *D*, create entries over time. The two team members interact when they read each others’ discussion entries. For each discussion entry, we collected (i) the ID of the team member creating it, (ii) the ID of the issue it belongs to, and (iii) the time of the entry’s creation. For reasons of confidentiality, we could not collect the content of the entries.

Together with three members of the product team, we further identified the following two practices (r1–r2) that allow us to obtain the interactions corresponding to the creation of each discussion entry:

r1 . Before team members write their *first* entry in a thread, they read the thread’s first entry to read the issue’s description. Additionally, they read the two most recent messages to learn about the current context of the discussion that their entry will continue.

r2 . For all *subsequent* entries, team members read the thread’s first entry to remind themselves of the issue. Additionally, they read every entry posted since (and including) their previous discussion entry.

Team member *D* reading a discussion entry of member *S* is equivalent to information flowing from *S* to *D*. Hence, we model all interactions derived from practices r1–r2 as directed links between the author and the reader of a discussion entry. We illustrate this in Fig 1a, where, for clarity, only the extracted links for *D* are shown. To capture the collaboration within the entire team, we aggregate the interactions as yearly directed multi-edge networks.

#### 3.2.2 Code review platform

To resolve issues, team members need to develop, review, and ultimately integrate *changes* to the codebase. This process is tracked and managed on the code review platform Aegis [47]. For Aegis, we again mined all actions of team members related to all changes. Specifically, we extracted (i) the ID of the team member performing an action, (ii) the ID of the corresponding change, (iii) the time at which the action was performed, and (iv) the type of the action. The possible types of actions are development *D*, review *R*, and integration *I*. The developer, reviewer, and integrator of a change must be different team members. In the ideal case, a change is first developed, then positively reviewed, and finally successfully integrated. However, both review and integration can fail, requiring further development and, hence, resulting in more complex action sequences (see Fig 1b for an example).

The change development process requires extensive interactions between team members that can be derived from the recorded actions following practices r3–r7, visualised in Fig 1b:

r3 . A reviewer *R* discusses the review’s outcome with the developer *D* of the change.

r4 . An integrator *I* discusses the integration’s outcome with the developer *D* of the change.

r5 . If the integration fails, the integrator *I* further discusses the detected problems with the reviewer *R* that positively reviewed the change.

r6 . A developer *D* that continues development after a failed review or integration discusses with the corresponding previous reviewer *R* or integrator *I*.

r7 . If a developer *D*_2_, reviewer *R*_2_, or integrator *I*_2_ take over from a previous developer *D*_1_, reviewer *R*_1_, or integrator *I*_1_, a handover discussion takes place.

As all discussions resulting from r3–r7 are bi-directional, we model them as undirected links between the involved team members. Aggregating all interactions within one year, yields yearly undirected multi-edge collaboration networks.

#### 3.2.3 Version controll system

From the Git-based version control system, we obtain interactions by extracting co-editing relations using the Python package git2net [48]. Motivated by the finding that a significant proportion of coordination between developers occurs via the code base [49], specifically when editing the same code [50], co-editing relations link team members consecutively modifying the same line of code. The links are directed following the arrow of time and time-stamped according to the time of the edit. We obtain yearly directed multi-edge networks by aggregating all co-editing relations for each year. As for the issue tracker and the code review platform, in these networks, team members are represented by their pseudo-anonymised ID.

## 4 Interaction structure detection

**Fig 2:**
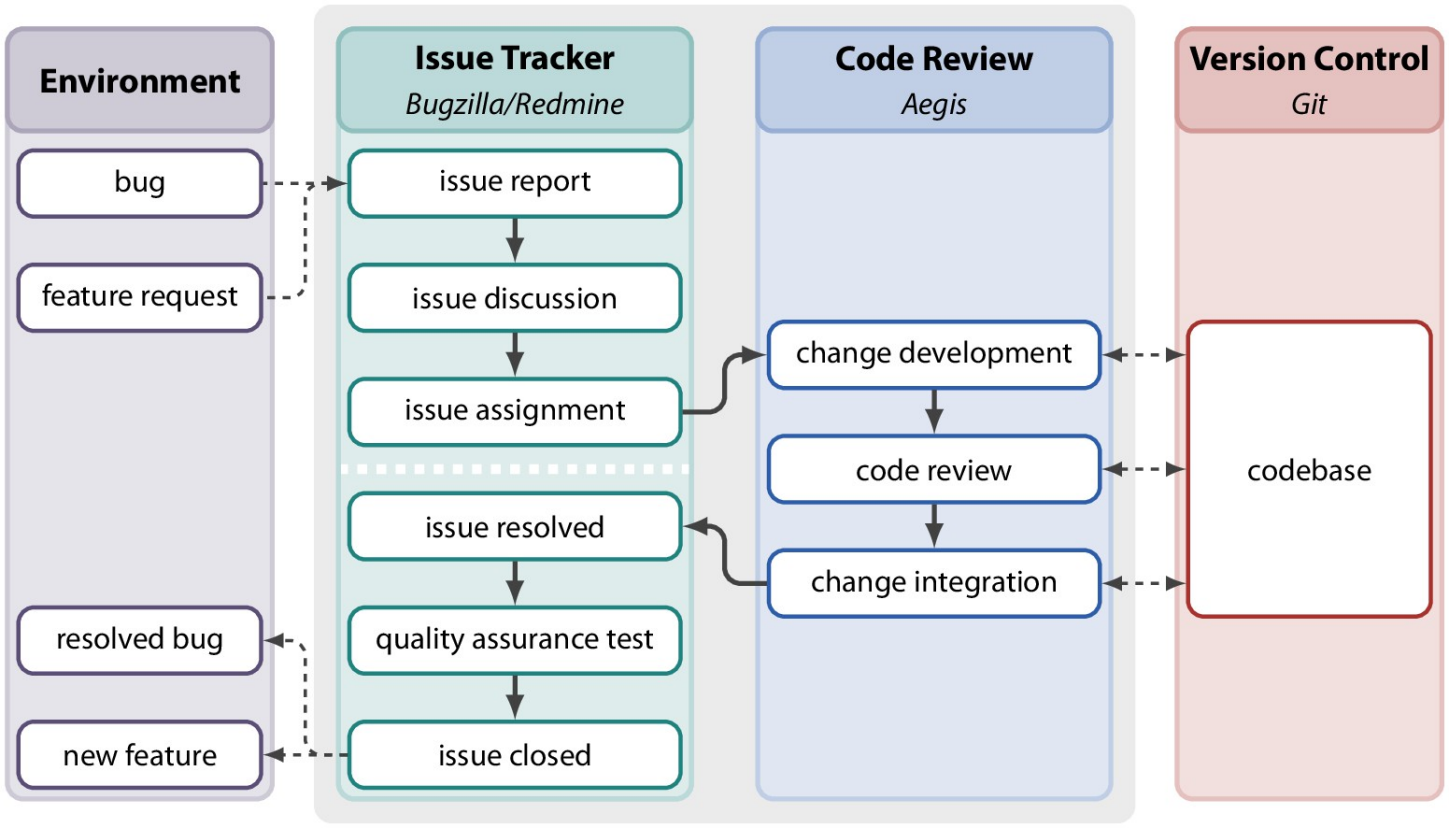
Representation of the process to resolve a typical issue in the product team at *genua*. The process takes place across three development platforms: the issue tracker, the code review platform, and the version control system. Backwards loops have been omitted for clarity.

In the previous section, we discussed the activities of a product team at *genua* over nine years. The primary operating mode of the team is to develop new features and fix bugs—collectively referred to as *issues*—in the product. To achieve this, the team uses three development platforms: an issue tracker, a code review platform, and a version control system. In Fig 2, we show the development process of a feature from the issue tracker to the code review platform and the version control system.

For each platform, we derived interaction networks aggregating the team’s activities for each year. In these interaction networks, team members are represented as nodes, and we identified each member’s role as a Developer, Stakeholder, Product Owner, or Documenter for each year. Interactions between different team members are represented by (multi-)edges between their corresponding nodes. For the purposes of our study, we treat all three platforms as equally important as they are all essential for the team’s development process. As we discuss in Section 8, this assumption is in line with the team’s perception of the platforms’ importance. However, when applying our proposed methodology to other teams, the importance of the platforms might differ, and the methodology should be adapted accordingly.

Our goal is to gain insight into the functional interaction structure of the team as a whole. To accomplish this, we require an aggregated representation of the team’s activities that captures the interaction preferences between members of different *roles* for the full development process. This means that we need to aggregate the interactions between individual team members based on their roles and combine the results across the different development platforms. This aggregated representation then enables us to study the team’s interactions on a holistic level, which will provide insights into how different functions within the team collaborate to develop the product.

### 4.1 Interaction count approach

A natural approach to aggregate the interactions between team members of different roles is to count how often these interactions occur. In mathematical terms, we count the number of edges between roles and collect these in a matrix *E*, whose elements *e*_*rs*_ are computed as follows:

$$\begin{array}{c} e_{rs}\left(A\right)=\sum_{i\in r}\sum_{j\in s}a_{ij} \end{array}$$ (1)

Here, *a*_*ij*_ are the elements of the interaction network’s adjacency matrix *A*, and r,s∈R are two roles. With *i* ∈ *r*, we iterate over all team members with role *r*. In the following, we refer to this method as the *interaction count approach*.

**Fig 3:**
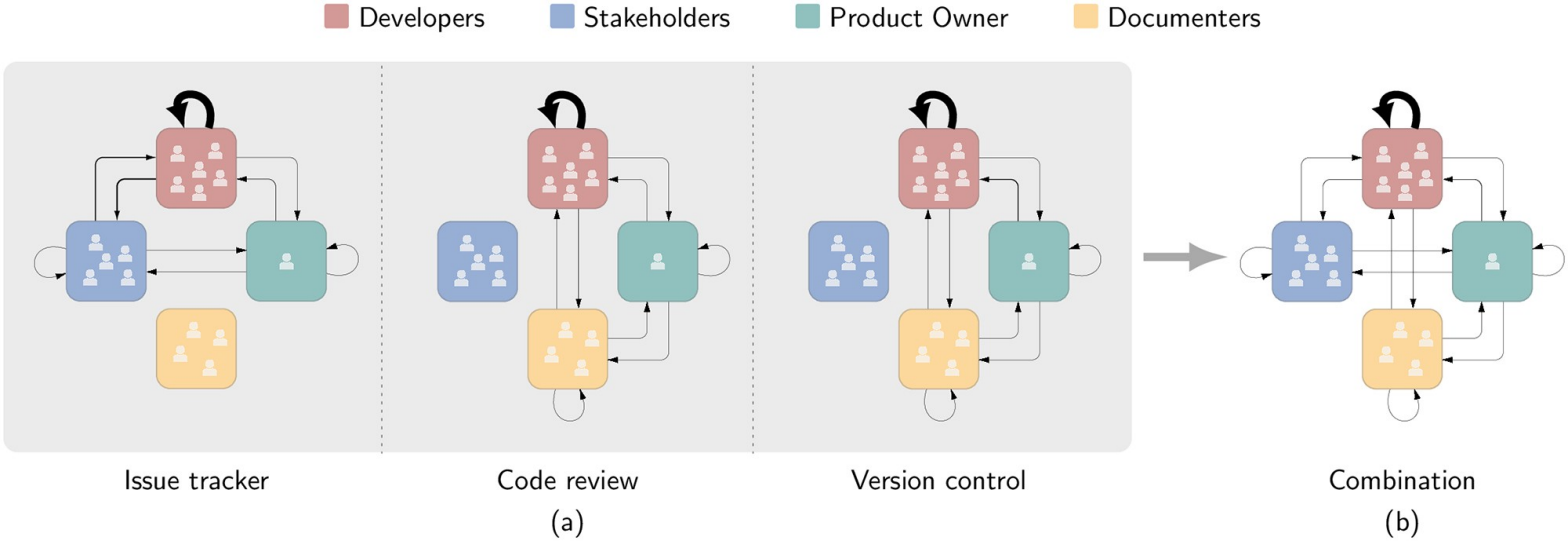
Interaction structure extracted using the interaction count approach. Nodes represent the four roles, and the width of links is proportional to the respective number of interactions between 2010 and 2018. The link widths indicate that except for interactions among Developers, all interaction counts are low and in the same order of magnitude. (a) Interaction structure for each platform. (b) Combined interaction structure for the three platforms.

In Fig 3a, we show the results of the interaction count approach for the different development platforms used by the product team at *genua*. We find that the results across the three platforms are remarkably similar. On each platform, one role is inactive, and all other roles form a fully connected network. All resulting aggregated networks are fully connected with the exception of one role that is inactive. Documenters are inactive on the issue tracker but instead track the changes in the documentation entirely via the code review platform. Similarly, Stakeholders interact on the issue tracker but do not appear on the code review and version control platforms. In addition, we find that Developers interact significantly more than all other roles. This finding persists across the three platforms and is shown by the width of links, which is proportional to the interaction count between the roles.

We show the combined activity across the three development platforms in Fig 3b, which we obtained as the average interaction count between the different roles. Fig 3b further highlights the absence of interactions between Stakeholders and Documenters who do not collaborate on any development platform. However, the high activity level of Developers far surpasses all other roles, concealing the nuances in their interaction counts. Thus, accounting for the different activity levels of the roles is necessary to gain a comprehensive understanding of their interaction preferences. Instead, based on the interaction count approach alone, we are unable to obtain any detailed insights.

### 4.2 From interaction counts to interaction preferences

As we found in the previous section, the interaction count approach does not account for the different activity levels of the roles. Specifically, it disregards:

(i) how many interactions each *role* can initiate and receive;
(ii) how many interactions each *individual* within a role can initiate and receive.

**Fig 4:**
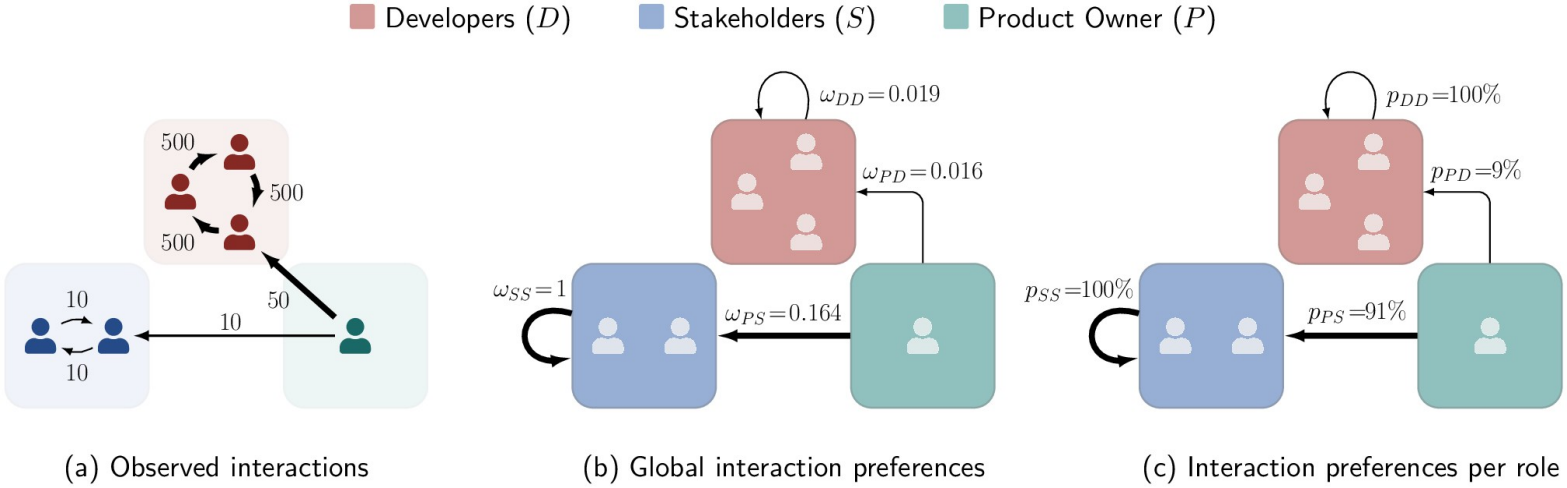
Applying our block model approach to extract the functional interaction structure in a synthetic example. (a) Counts of observed interactions between six team members belonging to three roles. (b) Global interaction propensities calculated for these observation counts. (c) Normalised interaction propensities for each role.

To highlight the consequences of this, we consider the synthetic example with six individuals from three roles shown in Fig 4a. The example assumes two Stakeholders interacting among themselves with moderate activity and three Developers interacting among themselves but with high activity. In addition, a Product Owner coordinates between the two groups. This Product Owner interacts 50 times with Developers but only ten times with Stakeholders. Does this mean that the Product Owner has an interaction preference towards Developers? While the interaction count approach would suggest this, we argue that it is not. Developers appear in 1550 interactions in total, whereas Stakeholders appear in *only* 30 interactions. The Product Owner is involved in ten of these 30 interactions. This means that the Product Owner is involved in around 3% of the Developers’ interactions but in 33% of the Stakeholders’ interactions, which suddenly seems like a lot.

### 4.3 Stochastic block model

Our example above illustrates the importance of considering the differences in activity levels among roles to accurately infer their interaction preferences. A popular approach used in social network analysis to incorporate such activity levels is the *stochastic block model (SBM)* [51]. The SBM assumes that the interaction probability between two team members can be determined solely based on their roles. Given an interaction network, the SBM first subdivides the team members according to their roles. Then, it computes the probability *b*_*rs*_ that two members of roles r,s∈R interact using a maximum likelihood approach:

$$\begin{array}{c} b_{rs}\left(E\right)=\frac{e_{rs}}{{\tilde{e}}_{rs}} \end{array}$$ (2)

Here, *e*_*rs*_ captures the number of multi-edges observed between roles r,s∈R. These counts are normalised by the number of *possible* multi-edges ${\tilde{e}}_{rs}$, accounting for the roles’ unique activity levels.

To get an intuition of how ${\tilde{e}}_{rs}$ is computed, we consider a simple case without multi-edges and with undirected interactions. If, for this case, we want to compute ${\tilde{e}}_{rs}$ for a role *r* with three members and a role *s* with two members, we count the number of different interactions that would be possible. Here, each of the three members of *r* could interact with each of the two members of *s*, resulting in ${\tilde{e}}_{rs}=3\cdot 2=6$ possible interactions. For a detailed explanation of how this can be extended to multiple directed interactions, we refer to the recent review [52].

**Fig 5:**
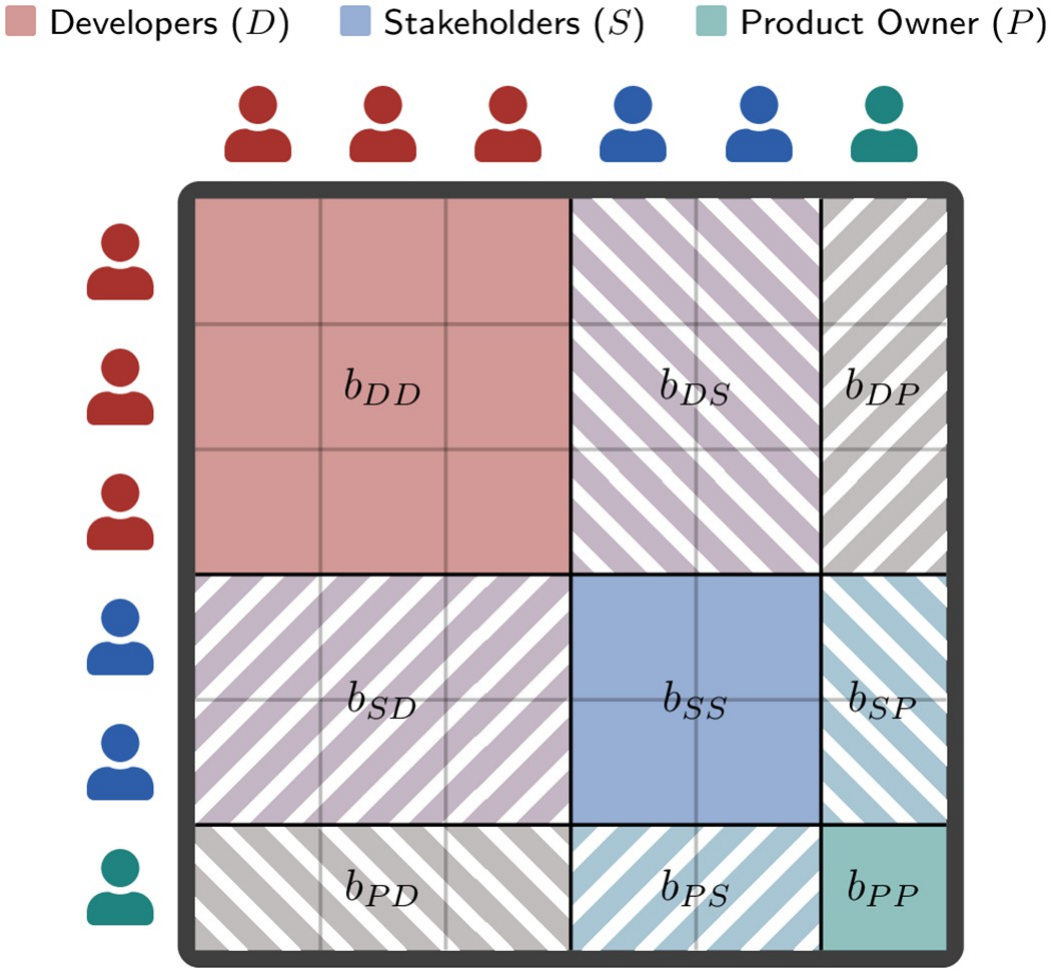
Illustration of the matrix *B* of an SBM for six individuals from three roles R={D,S,P}. The SBM subdivides the inviduals into three groups according to their role. For each pair of roles, r,s∈R, the SBM then computes a single interaction probability *b*_*rs*_.

We show the individual interaction probabilities *b*_*rs*_ collected in a matrix *B* for an example with three roles, *r*, *s* ∈ {*D*, *S*, *P*} in Fig 5. The SBM can be efficiently fit to empirical data and is well-suited to capture the modular structure of real-world networks, explaining its popularity. However, while it accounts for the differences in activity levels *among* roles, it does not capture differences in activity levels among members *within* each role. Thus, the SBM satisfies requirement (i) derived in Section 4.2, but falls short of fulfilling requirement (ii).

### 4.4 Block-constrained configuration model

To account for *both*, the activity levels of roles *and* individuals, we propose to use the *block-constrained configuration model* (BCCM) [24]. The BCCM is a flexible network-theoretic tool to study the structure of networks while accounting for heterogeneity in the activity on the level of nodes—i.e., team members. To achieve this, the BCCM introduces a matrix Ξ. The elements *ξ*_*ij*_ of Ξ estimate the number of possible interactions (multi-edges) that a team member *i* can initiate with a team member *j* based on their respective out- and in-degrees $d_{i}^{\text{out}},d_{j}^{\text{in}}$:

$$\begin{array}{c} \xi_{ij}=d_{i}^{\text{out}}\cdot d_{j}^{\text{in}} \end{array}$$ (3)

Based on Ξ and the interaction counts *E*, the BCCM then computes interaction propensities (We will further comment on the differences between interaction probabilities and propensities in Section 4.5.):

$$\begin{array}{c} \omega_{rs}\left(E,\Xi \right)=-\text{log}(1-\frac{e_{rs}}{\sum_{i\in r}\sum_{j\in s}\xi_{ij}}) \end{array}$$ (4)

To determine *ω*_*rs*_ for a given interaction network, we use the function bccm in the R library ghypernet [53].

Although the *ω*_*rs*_ of the BCCM (cf. Eq (4)) and the *b*_*rs*_ of the SBM (cf. Eq (2)) are similar, they differ in the way interaction counts between roles *e*_*rs*_ are normalised. The SBM only takes into account activity levels of roles by normalising by ${\tilde{e}}_{rs}$, whereas the BCCM sums the individual *ξ*_*ij*_ of all members of roles *r* and *s*. In doing so, the BCCM accounts for the activity level of individual team members and satisfies requirement (ii) from Section 4.2.

### 4.5 From propensities to interaction probabilities

Both the interaction counting approach and the SBM yield *probabilities*, which can be directly interpreted as interaction preferences between roles. Instead, the BCCM yields interaction *propensities*. We now discuss how we can obtain interaction preferences from such propensities based on the synthetic example introduced in Section 4.2. The interaction network for this example is shown in Fig 4a. We remind that in this example we have two Stakeholders and three Developers that interact among each other with moderate and high frequencies, respectively. A Product Owner coordinates with the two groups interacting five times more frequently with Developers compared to Stakeholders. For this scenario, the interaction counting approach suggested a strong interaction preference of the Product Owner towards Developers. However, our discussion in Section 4.2 suggested the opposite, as the Product Owner is involved in 33% of the Stakeholders’ interactions but only 3% of the interactions of Developers.

In Fig 4b, we show the interaction propensities *ω* obtained from the BCCM approach for this example. The BCCM yields an interaction propensity of *ω*_*PS*_ = 0.164 between the Product Owner and Stakeholders. In contrast, despite the higher interaction count between the Product Owner and Developers, we obtain a ten-times lower interaction propensity of *ω*_*PD*_ = 0.016. Thus, the BCCM suggests that the Product Owner is ten times more likely to interact with Stakeholders than with Developers when taking into account that Stakeholders are less active than Developers.

In the example above, we have seen that the propensities obtained from the BCCM can only be interpreted *in relation to each other*. To allow for *individual interpretation*, we propose to normalise the propensities as:

$$\begin{array}{c} p_{rs}=\frac{\omega_{rs}}{\sum_{\rho \in R}\omega_{r\rho }}\in \left[0,1\right] \end{array}$$ (5)

The resulting *p*_*rs*_ can be interpreted as the probability of an individual with role *r* to interact with an individual with role *s*. As shown in Fig 4c, the normalisation retains the ten-to-one ratio in the Product Owner’s interaction preferences towards Stakeholders, but makes it immediately visible as *p*_*PS*_ = 91%.

## 5 Detecting *genua*’s interaction structure

**Fig 6:**
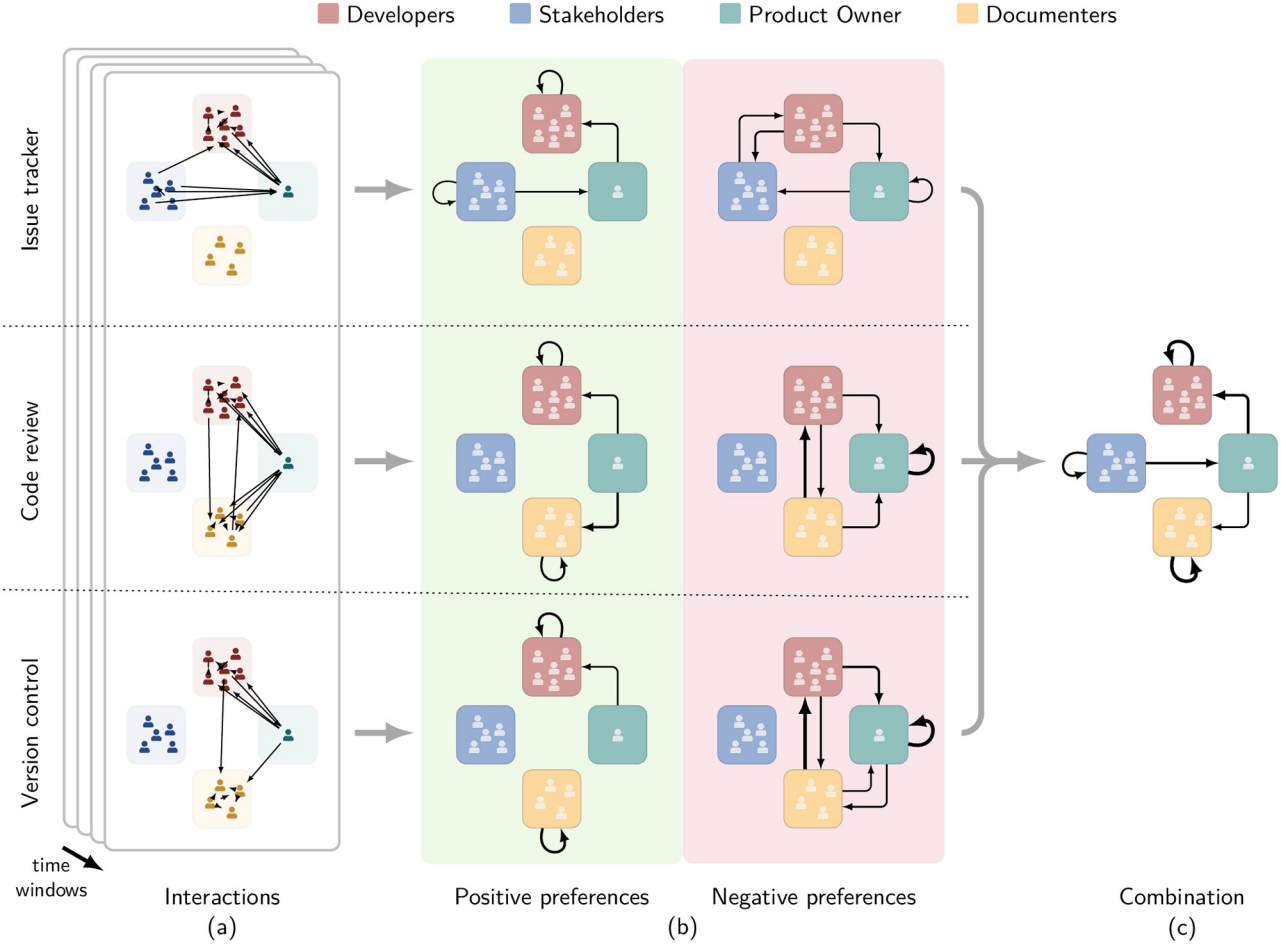
Applying our block model approach to extract the functional interaction structure for the studied project team at *genua*. (a) Interactions between team members represented as interaction networks for each platform and year between 2010 and 2018. (b) Interaction preferences computed separately for positive (>25%) and negative (< 25%) preferences and averaged across years. The width of links corresponds to the strength of the positive or negative preference. (c) The functional interaction structure of the team obtained as the combination of the positive preferences across the three platforms. By controlling for the activity of the team members, the block model approach allows for deeper insights going beyond the edge aggregation approach, which only identified two categories of links.

We now apply our BCCM approach to detect the functional interaction structure of the studied product team at *genua*. We visualise our approach in Fig 6. Based on the interaction networks (cf. Fig 6a) for multiple platforms and multiple years that we collected in Section 3, we first compute the interaction preferences separately for each platform and year before averaging them across the years.

The team at *genua* has members with four roles. To interpret the interaction preferences obtained by the BCCM, we compare them to a baseline in which we assume that members of all roles interact with equal probability. In other words, we compare the probabilities *p* from the BCCM to a baseline probability of 25% (The threshold of 25% assumes that interactions can occur between members of the same role. This is intuitively true for Developers, Stakeholders, and Documenters as there are always multiple active members of these roles. The Product Owner is a special case. While, in principle, there is only one Product Owner active at any point in time, our data contains multiple transitions between Product Owners, resulting in two Product Owners being recorded for a year. Therefore, we opted to treat Product Owners analogous to the other roles and did not introduce an exception.). We interpret observed interaction probabilities above this baseline as *positive* interaction preferences between the roles. Instead, we interpret probabilities below the baseline as *negative* interaction preferences. We show the resulting positive and negative interaction preferences in Fig 6b.

### 5.1 Positive interaction preferences

On the issue tracker, Stakeholders, the Product Owner, and Developers are active. The interaction preferences suggest that Stakeholders predominantly interact with themselves and the Product Owner. In turn, the Product Owner has an interaction preference towards Developers. Finally, Developers again preferentially interact among themselves.

The code review platform has activity from all roles other than Stakeholders. Here, the interaction preferences suggest interactions from the Product Owner towards both Developers and Documenters, who in turn show a preference to interact among themselves.

Finally, the version control system shows a similar pattern as the code review platform. Again, only the Product Owner, Developers, and Documenters are active. The Product Owner has an interaction preference towards Developers, and Developers and Documenters interact primarily among themselves. The only difference to the code review platform is the absence of a positive preference between the Product Owner and Documenters.

### 5.2 Negative interaction preferences

We visualise these negative interaction preferences in the right column of Fig 6b. The positive preferences discussed above imply the existence of corresponding negative preferences towards the other roles. This means that the structure of the negative preferences, i.e., which links exist, is complementary to the structure of the positive preferences. Therefore—rather than its structure—we are particularly interested in the *strength* of the negative preferences displayed by each role.

Stakeholders are only active on the issue tracker, where they have positive interaction preferences with themselves and the Product Owner. As Documenters are not active on the issue tracker, Stakeholders only have a negative interaction preference towards Developers.

The Product Owner is active on all platforms. As indicated by the thin width of the links, the Product Owner does not show strong negative interaction preferences to other roles. However, we find a self-loop suggesting that Product Owners interact significantly less than expected among themselves. This is intuitive, as there is only one Product Owner active at any point in time. Therefore, if we observe more than one Product Owner in one of our yearly snapshots, this indicates a transition between the two at some point during the year. However, as they are active consecutively and not simultaneously, we find fewer interactions than their activity suggests.

Developers are also active on all platforms. As indicated by the similar width of all edges from Developers to all other roles, Developers do not show strong negative interaction preferences towards any role.

Finally, Documenters are active on the code review and version control platforms. For Documenters, we find a strong negative interaction preference towards Developers.

So far, we have discussed interaction preferences for each platform separately. We now combine them to obtain the functional interaction structure of the team across all platforms. As the positive and negative interaction preferences are complementary, both reveal the same interaction structure. However, positive interaction preferences are more natural to interpret. Therefore, we show the functional interaction structure obtained by combining the positive interaction preferences in Fig 6c. Overall, we find that Stakeholders represent the input into the development team. Stakeholders interact primarily with the Product Owner who, in turn, has strong interaction preferences towards Developers and Documenters. These two roles represent sinks in the team, primarily interacting among their own role and not with each other.

## 6 Validating the detected interaction structure through interviews

We now validate the detected interaction structure and enrich it with information on the *function* of these relations—yielding the team’s organigraph. To this end, we conducted interviews with five experienced members of the studied product team (Int1—Int5). As interview participants, we selected three team members whom our analysis identified as central to the team’s development process. In addition, these team members (Int1–Int3) have been with the team for more than five years and have held different roles allowing them to provide a comprehensive view of the team’s development processes. In addition, we asked to interview two Stakeholders (Int4 and Int5) to gain insights into the team’s external interactions.

**Fig 7:**
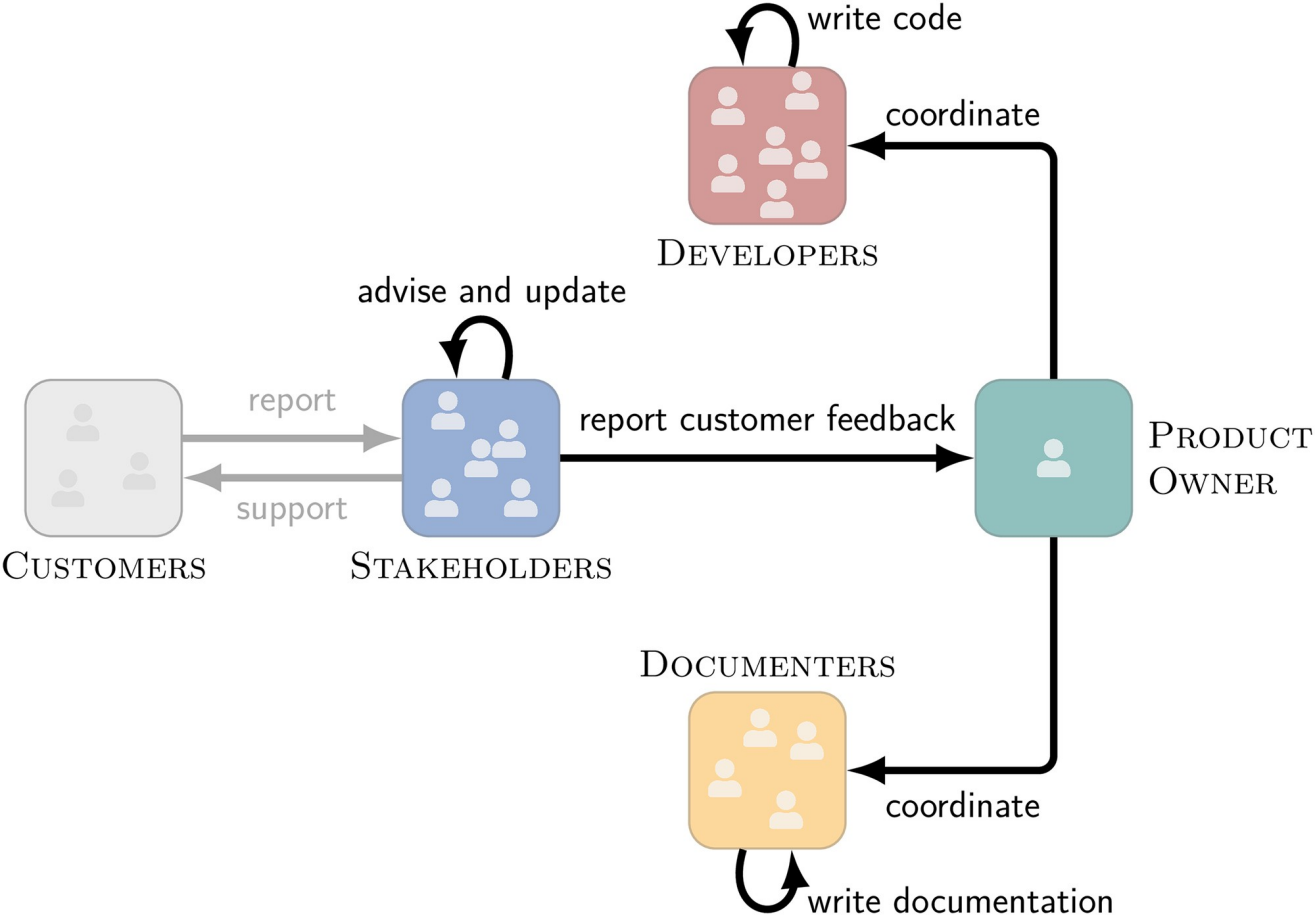
Organigraph of the product team at *genua* across all three development platforms. To obtain the organigraph, we start from the functional interaction structure detected using our block model approach (see Fig 6c). We then enrich it with the information collected during our interviews to allow for an interpretation.

Our set of interviewees (male, age range 30–50) consists of two Developers, two Stakeholders, and one former Product Owner, ensuring that we get a broad range of first-hand perspectives into the product team’s development processes. All interviews were held online in a video chat in March 2021 and lasted for approx. 60 minutes, followed by a debriefing. We set up each interview in a semi-structured format, combining closed-ended survey questions with open-ended discussions [54]. The interviews were conducted without the aid of any additional material. As we summarise in the following, the interviews validated and explained all interaction preferences identified in our quantitative analysis (cf. Section 4). We show the organigraph enriching our quantitative results with the explanations from the interviews in Fig 7.

### 6.1 The role of Stakeholders

As we discussed in Section 3.1, the majority of Stakeholders are customer-facing and often located at the customer’s sites. Consequently, they are the first to learn about new bugs, required features, or new use cases for which they forward feedback directly to the Product Owner.

“*The customers’ wishes for new features are supposed to be assigned to the* Product Owner.”

[Statement by Int3]

“Stakeholders *discuss new features always in direct coordination with the PO*.”

[Statement by Int4]

Simultaneously, the geographical distribution of Stakeholders explains their reduced interactions with Documenters and Developers.

“Stakeholders *are not at the company’s [i.e., genua’s] site, and therefore can’t just go into a* Developer*’s office and ask*.”

[Statement by Int4]

Further, Stakeholders do not have access to the code review and version control platforms, explaining the observed lack of interactions there.

“*Actually, normal* Stakeholders *have nothing to do with* Aegis *[the code review platform] and* Git *[the version control system]*.”

[Statement by Int3]

Internally, Stakeholders update and advise each other on common problems and critical bugs, which they then champion to be prioritised in the team’s weekly bug meetings.

### 6.2 The role of the Product Owner

Collecting the information from Stakeholders, the Product Owner leads the weekly bug meetings and is responsible for scheduling and prioritising what is being worked on.

“*In bug meetings, the* Product Owner, *some* Stakeholders, *and also a couple of* Developers, *who took care of the bugs, discuss prioritisation, and their initial analysis*.”

[Statement by Int1]

The Product Owner then coordinates and oversees the rest of the team. Thus, the Product Owner indeed acts as a fixed mediator for feedback from the Stakeholders to the Developers and Documenters, confirming the results of our quantitative analysis.

### 6.3 The roles of Developers and Documenters

Following the bug meeting, the Developers work on changes resolving the bugs or implementing the discussed features and Documenters update the product’s documentation accordingly.

“*Based on the outcome of the bug meeting, the* Developers *develop. And the* Documenters *can, of course, also see what is written and then document this*.”

[Statement by Int1]

All interviewees agreed that these two processes occur mostly independently, explaining the infrequent interactions between Developers and Documenters.

“Documenters *and* Developers *have their closed problem domains. The* Developer *tries to get a feature working from a technical perspective, and the* Documenter *tries to explain it to a user at the other end*.”

[Statement by Int5]

However, interactions within their own role still occur very frequently. One key reason for this is *genua*’s internal review process, which requires all changes—including changes to the documentation—to be developed, reviewed, and integrated by three different team members, which automatically sparks interactions between many different members.

“*Whenever something is changed, someone has to look at it [i.e., review and integrate it into the codebase]*.”

[Statement by Int5]

In summary, with our interviews, we could validate and explain all detected interaction preferences—both positive and negative—between roles. This validates our quantitative approach and shows that we can extract the functional interaction structure of teams accurately and in a computationally efficient manner.

## 7 Interaction structure optimisation

In Section 4, we started our analysis of the product team from the observed individual interactions between team members. We then grouped the team members according to their role in the team, yielding the functional interaction structure capturing the interaction preferences between members of different roles reported in Fig 6c. Through this step, we aggregated all team members of a given role into a single representative node in the resulting role interaction network. Implicitly, this assumes that all members of a role are similar to the degree that they can be considered as interchangeable. This strong assumption is unlikely to be fully fulfilled in any real-world organisation (cf. Section 2 for a discussion). The role definitions from Section 3.1 already state that, e.g., Stakeholders take on multiple different functions and specialisations. Similarly, our interviews also suggest a degree of heterogeneity amongst Developers, both in terms of their experience and their knowledge of different parts of the codebase.

“*I think there are still comfort zones where people make initial changes and whom you let do it [make changes in a specific area of the codebase]*.”

[Statement by Int5]

However, by employing agile development methods, the team actively aims to promote and enhance knowledge diffusion among the Developers, to reduce the risk of knowledge loss when a member leaves the team.

“*One of the philosophies of Scrum is that ‘everyone can do everything’ to address precisely the problems arising when the bus comes [referring to the truck factor, which is also known as the bus factor], or Google simply pays more. Thus we try to counteract exactly these problems in advance through XP [Extreme Programming] and pair programming [deliberate pairing of team members with different expertise]*.”

[Statement by Int1]

This motivates a final experiment in which we use our extracted interaction preferences to assess where the team currently stands and to what extent further homogeneity among the members of roles could improve knowledge diffusion within the team.

To quantify knowledge diffusion, we use the measure *potentiality* (Pot) proposed in [29]. Potentiality utilises the notion of entropy to quantify the extent to which members distribute their interactions across the entire team rather than among a few specific collaborators:

$$\begin{array}{c} \text{Pot}≔\frac{H^{\text{observed}}}{H^{\text{max}}}\in \left[0,1\right]. \end{array}$$ (6)

Here, *H*^observed^ is the entropy of the observed interaction distribution, and *H*^max^ is the highest possible entropy achieved when all team members interact with everyone else equally often. A potentiality close to 1 indicates that most members interact with the entire team, whereas a potentiality close to 0 indicates that many members only have a few interaction partners.

As we discussed in Section 4.4, we encode the team’s interaction *structure* through the *ω* parameters of the corresponding BCCM (cf. Eq (4)). In contrast, potentiality is computed on interaction *networks*. Given an interaction structure—i.e., a specified BCCM—we obtain the distribution of likely interaction networks using the sampling approach implemented in [53]. Subsequently, we compute potentiality for all sampled networks obtaining a distribution of values capturing—and hence allowing us to compare—the team’s knowledge diffusion for different interaction structures.

**Fig 8:**
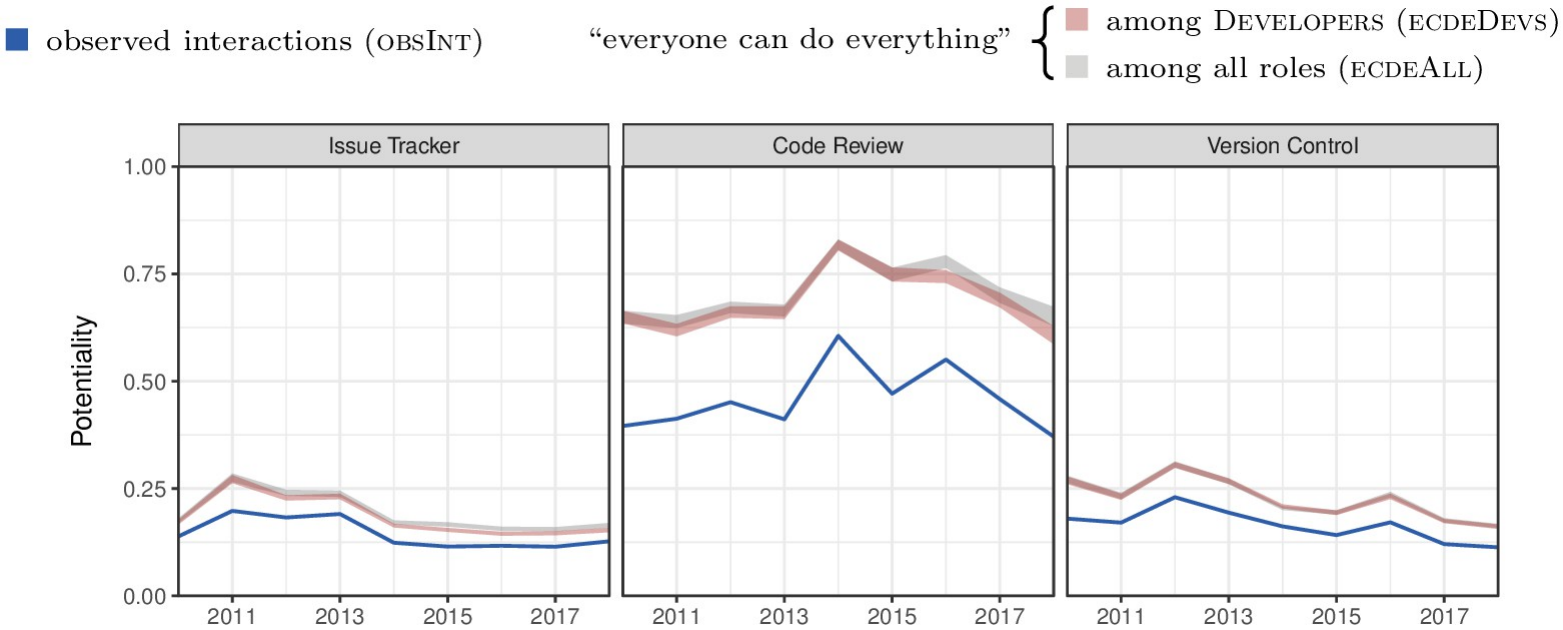
Potentiality as a measure for knowledge diffusion. We compare the observed interactions (obsInt) against two synthetical benchmark scenarios proposed by *genua*. The first scenario (ecdeDevs) assumes homogeneity among all Developers. The second scenario (ecdeAll) assumes homogeneity among members of all roles. We show the results for all years and development platforms separately. The team shows knowledge diffusion in the upper third of the attainable range defined by the benchmark scenarios. The two benchmark scenarios result in almost identical levels of knowledge diffusion.

We report our results in Fig 8. In ■, we show the potentiality computed for the observed interactions (obsInt) over time. We compare the knowledge diffusion in the observed case to two synthetically created benchmark scenarios suggested by *genua*. In the first scenario (ecdeDevs), shown in ■, we assume that the team achieves the stated aim that “everyone can do everything” (ecde) among Developers, effectively making them interchangeable. This corresponds to a BCCM model where all developers are aggregated into a single block, while all other team members are represented by individual blocks. Finally, in the second scenario (ecdeAll) shown in ■, we assume that “everyone can do everything” holds not only for Developers but for all roles. This corresponds to a BCCM where, analogous to the organigraph in Fig 7, all team members are aggregated into four blocks corresponding to their role.

For all platforms and all years we observe Po(ecdeAll) ≥ Pot(ecdeDevs) ≥ Pot(obsInt). This ordering aligns with our expectation that knowledge diffusion has an inverse relation to the heterogeneity of members of a role.

Notably, the difference between ecdeDevs and ecdeAll is always diminishingly small. This means that almost all possible improvements in knowledge diffusion can already be achieved if “everyone can do everything” among Developers. As indicated by obsInt, the team currently achieves a knowledge diffusion corresponding to around 70% of the optimal case ecdeAll. Our analysis suggests that to improve this further, the team should target knowledge diffusion among Developers first.

Comparing the three platforms, we observe significantly higher knowledge diffusion on the code review platform. This indicates that *genua*’s efforts to promote interactions by requiring that at least three different team members contribute to all changes are successful. Finally, the code review platform is primarily used by Developers, explaining why the difference between obsInt and ecdeDevs is largest here.

In conclusion, we find that the studied team achieves knowledge diffusion in the upper third of the attainable range. Our analysis shows that almost the entire remaining gain can already be achieved by obtaining optimal knowledge diffusion among developers (cf. ecdeDevs). Working towards this, the team at *genua* implements various agile methods, including Scrum, Extreme Programming, and pair programming.

## 8 Threats to validity

Our study is subject to some threats to validity, which we discuss in the following.

### 8.1 Internal validity

While we have taken the utmost effort and care to obtain complete and correct data on the interactions among all members of the product team, there are three limitations that we discuss in the following.

First, for our study, we mined all actions logged in the complete databases of all three development platforms used by the analysed team. From our discussions with team members, we learned that no development occurs without generating entries on these platforms as the team strictly enforces all bugs and feature requests to be tracked and version controlled. That said, due to confidentiality concerns, we could not obtain and analyse any text data. Next to the content of the interactions on the three development platforms, this also means that we did not have access to any email or chat communication. Finally, interactions such as personal discussions are not recorded. As a consequence, these interactions are missing from our data.

Second, as discussed in Section 3.2, the development platforms record actions instead of interactions between team members. In discussion with members of the product team, we identified a set of practices allowing us to extract the interactions corresponding to the recorded actions. However, we expect a degree of heterogeneity in the behaviour of team members, which is not covered by the practices. Furthermore, we expect team members to adapt their behaviour over time and based on the context of the situation, resulting in changes over time.

Finally, the three development platforms record different types of interactions (discussions, code reviews, and co-editing of code). In our interviews, we discussed the possibility of weighing the different types of interactions for our combined results. However, there was no consensus among our interviewees concerning which platform is most important for them. Therefore, for our combined results, we treated all platforms as equal.

### 8.2 Construct validity

In this study, we aimed to extract the functional relations between roles in a product team. To this end, we studied the team’s interactions using a block model approach. The resulting relations match those identified in our subsequent interviews, confirming the usefulness of our approach. However, we cannot entirely rule out unlikely cases in which our approach missed relations that none of the five interviewed team members was aware of, as those would show up neither in our data nor the interviews.

In the second part, we used the resulting functional interaction structure to assess knowledge diffusion in the team. To this end, we used the entropy-based measure *potentiality*. While our results suggest that potentiality captures knowledge diffusion adequately, additional measures, e.g., also capturing the content of interactions, could further improve our analysis. Unfortunately, as we did not have access to any text data, we could not further explore this.

Finally, for our two hypothetical benchmark scenarios ecdeDevs and ecdeAll, we assumed perfect homogeneity among all team members of a role. We argue that the scenarios are helpful as optimal cases the team can work towards. However, different activity levels of team members, turnover, and differences in team members’ experience with the product will always cause the scenarios to remain purely theoretical. In addition, our analysis excluded the discussion of the benefits of heterogeneity, e.g., increased productivity and creativity [55, 56], which we will assess in future work.

### 8.3 External validity

Lastly, we performed our analysis in a case study for a single product team at *genua*, which sparks questions regarding the external validity of our analysis. Our analysis approach solely relies on time-stamped interaction data and information regarding team members’ roles and makes no assumptions concerning their content. Therefore, we do not see concerns regarding the generalisability of our approach.

## 9 Conclusions

An efficient interaction structure facilitates knowledge diffusion, allowing the team to maintain its performance and retain its knowledge base even when team members leave. However, particularly in flexible, self-organised teams, the interaction structure evolves over time. While this allows the team to adapt to new challenges, it also bears the risk of undesirable outcomes, such as reduced software quality or the emergence of community smells.

This paper showed how the functional interaction structure, i.e., the preferences with which members of different roles interact with each other, can be directly inferred from the traces that team members create on their development platforms during their daily work. We began by comparing three methods of aggregating interactions between team members based on their roles. Our findings revealed that if all team members and roles demonstrate equal activity levels, a reliable representation of the team’s functional interaction structure can be attained by merely counting the interactions between the roles. However, when there are variations in activity levels, a statistical model is necessary. To this end, the stochastic block model is a well-established approach that can consider differences in the activity levels of roles. However, it cannot account for variations in activity levels between team members. To address this issue, we proposed to use the block-constrained configuration model to detect a team’s functional interaction structure. Our approach further provides a more comprehensive understanding of positive and negative interaction preferences by comparing this functional interaction structure to a random baseline.

In a case study, we mined comprehensive data tracking the development process of a product team at the German IT security company *genua GmbH* across three development platforms. We then applied our approach to extract the functional interaction structure of the team. We conducted semi-structured interviews with five team members in which we validated the accuracy of the detected interaction structure. In addition, the interviews allowed us to enrich the detected interaction structure with information on the purpose of each interaction. This made the interaction structure interpretable and yielded the team’s organigraph.

During the interviews, we further learned that to prevent knowledge loss, *genua* strives for homogeneity among members of a role—i.e., “everyone can do everything” across members of a role. This motivated a final experiment in which we showed how our approach enables teams to compare themselves against synthetic benchmark scenarios. Specifically, we studied the knowledge diffusion in the development team and compared it to two scenarios suggested by *genua*. The first scenario assumed homogeneity only among Developers, while the second scenario assumed homogeneity for all roles. Our results demonstrated that the team currently reaches knowledge diffusion in the upper third of the attainable range. We further showed that reaching homogeneity for all roles in the team is not required. Instead, almost all possible gains in knowledge diffusion can already be achieved by further promoting interactions between Developers, which the team does by applying Scrum, Extreme Programming, and pair programming.

Our approach is computationally efficient, allowing teams to track the results of their efforts and manage their interaction structure in real-time, based solely on readily available development data.
